# Comparison of Two Multilocus Sequence Based Genotyping Schemes for *Leptospira* Species

**DOI:** 10.1371/journal.pntd.0001374

**Published:** 2011-11-08

**Authors:** Ahmed Ahmed, Janjira Thaipadungpanit, Siriphan Boonsilp, Vanaporn Wuthiekanun, Kishore Nalam, Brian G. Spratt, David M. Aanensen, Lee D. Smythe, Niyaz Ahmed, Edward J. Feil, Rudy A. Hartskeerl, Sharon J. Peacock

**Affiliations:** 1 WHO/FAO/OIE and National Collaborating Centre for Reference and Research on Leptospirosis, Department of Biomedical Research, Royal Tropical Institute (KIT), Amsterdam, The Netherlands; 2 Mahidol-Oxford Tropical Medicine Research Unit, Faculty of Tropical Medicine, Mahidol University, Bangkok, Thailand; 3 Medical Proteomics Unit, Office for Research and Development, Faculty of Medicine Siriraj Hospital, Bangkok, Thailand; 4 Pathogen Biology Laboratory, Department of Biotechnology, School of Life Sciences, University of Hyderabad, Hyderabad, India; 5 Department of Infectious Disease Epidemiology, Imperial College London, London, United Kingdom; 6 WHO/FAO/OIE Collaborating Centre for Reference and Research on Leptospirosis, Centre for Public Health Sciences, Queensland Health Scientific Services, Brisbane, Australia; 7 Institute of Biological Sciences, Universiti Malaya, Kuala Lumpur, Malaysia; 8 Department of Biology and Biochemistry, University of Bath, Bath, United Kingdom; 9 Department of Microbiology and Immunology, Faculty of Tropical Medicine, Mahidol University, Bangkok, Thailand; 10 Department of Medicine, University of Cambridge, Addenbrooke's Hospital, Cambridge, United Kingdom; Institut Pasteur, France

## Abstract

**Background:**

Several sequence based genotyping schemes have been developed for *Leptospira* spp. The objective of this study was to genotype a collection of clinical and reference isolates using the two most commonly used schemes and compare and contrast the results.

**Methods and Findings:**

A total of 48 isolates consisting of *L. interrogans* (n = 40) and *L. kirschneri* (n = 8) were typed by the 7 locus MLST scheme described by Thaipadungpanit et al., and the 6 locus genotyping scheme described by Ahmed et al., (termed 7L and 6L, respectively). Two *L. interrogans* isolates were not typed using 6L because of a deletion of three nucleotides in *lipL32*. The remaining 46 isolates were resolved into 21 sequence types (STs) by 7L, and 30 genotypes by 6L. Overall nucleotide diversity (based on concatenated sequence) was 3.6% and 2.3% for 7L and 6L, respectively. The D value (discriminatory ability) of 7L and 6L were comparable, i.e. 92.0 (95% CI 87.5–96.5) vs. 93.5 (95% CI 88.6–98.4). The dN/dS ratios calculated for each locus indicated that none were under positive selection. Neighbor joining trees were reconstructed based on the concatenated sequences for each scheme. Both trees showed two distinct groups corresponding to *L. interrogans* and *L. kirschneri*, and both identified two clones containing 10 and 7 clinical isolates, respectively. There were six instances in which 6L split single STs as defined by 7L into closely related clusters. We noted two discrepancies between the trees in which the genetic relatedness between two pairs of strains were more closely related by 7L than by 6L.

**Conclusions:**

This genetic analysis indicates that the two schemes are comparable. We discuss their practical advantages and disadvantages.

## Author Summary

Two independent multilocus sequence based genotyping schemes (denoted here as 7L and 6L for schemes with 7 and 6 loci, respectively) are in use for *Leptospira* spp., which has led to uncertainty as to which should be adopted by the scientific community. The purpose of this study was to apply the two schemes to a single collection of pathogenic *Leptospira*, evaluate their performance, and describe the practical advantages and disadvantages of each scheme. We used a variety of phylogenetic approaches to compare the output data and found that the two schemes gave very similar results. 7L has the advantage that it is a conventional multi-locus sequencing typing (MLST) scheme based on housekeeping genes and is supported by a publically accessible database by which genotypes can be readily assigned as known or new sequence types by any investigator, but is currently only applicable to *L. interrogans* and *L. kirschneri*. Conversely, 6L can be applied to all pathogenic *Leptospira* spp., but is not a conventional MLST scheme by design and is not available online. 6L sequences from 271 strains have been released into the public domain, and phylogenetic analysis of new sequences using this scheme requires their download and offline analysis.

## Introduction

Leptospirosis is a common zoonotic disease worldwide, with a particularly high prevalence in warm humid countries [1]–[4]. About 350,000 severe cases of leptospirosis are estimated to occur annually, with case fatality reports up to 50% [5]–[7]. Reported cases are likely to be a gross under-estimate of global incidence rates, the result of a combination of factors including lack of surveillance, diagnostics and notification in those countries with the highest disease burden. Leptospirosis is currently considered a globally re-emerging disease, with frequent outbreaks in South East Asia (including Thailand, India, The Philippines and Sri Lanka) as well as in Latin America [3], [8]–[14]. International travel also leads to presentation of leptospirosis cases in settings where incidence is low and clinicians are unfamiliar with its clinical manifestations [7],[15].

Identification and typing of *Leptospira* species plays an important role in understanding disease epidemiology and pathogenicity, together with the development of diagnostic tools, effective vaccines and preventive strategies. During the last three decades many molecular typing methods have been proposed for *Leptospira* spp. These include DNA-DNA hybridization analysis [16]–[19], randomly amplified polymorphic DNA (RAPD) fingerprinting [20], arbitrarily primed PCR (AP-PCR) [21], [22], pulsed field gel electrophoresis (PFGE) [23], [24], restriction fragment length polymorphism (RFLP) analysis [25], [26], bacterial typing methods based on insertion sequences (IS) [27], detection of variable number of tandem repeats (VNTR) [28], [29], *rrs* sequencing [30]–[32], and sequencing of specific genes or gene fragments including *rpoB*, *gyrB*, *secY* and *ligB* [33]–[37].

Multilocus sequencing typing (MLST) has been widely adopted for the study of bacterial evolution and population biology of a large number of microbial species [38], and represents the leading molecular method for bacterial genotyping. MLST based on 7 housekeeping loci has been developed for *Leptospira* [39], and is supported by a publically accessible database by which genotypes can be readily assigned as known or new sequence types. An alternative sequence based genotyping scheme of 6 loci including housekeeping genes, a 16S rRNA gene and genes encoding surface-expressed proteins has also been developed and used by several groups. This has led to uncertainty as to which scheme should be adopted. The aim of the current study was to compare the two schemes in terms of their discriminatory ability, both within and between species, by generating data using both schemes for a single set of isolates. We also discuss the practical aspects relating to each scheme.

## Materials and Methods

### *Leptospira* isolates and DNA isolation

The *Leptospira* isolates used in this study and their providers are shown in Table 1. Genomic DNA was extracted from laboratory bacterial cultures as described previously [39], [40].

**Table 1:** *Leptospira* isolates used in this study.

Species	Serovar	Strain	ST (7 loci scheme)#	Origin	Source*
*L. interrogans*	Copenhageni	M 20	17	Reference	Aus& KIT
*L. interrogans*	Guaratuba	An 7705	37	Reference	Aus
*L. interrogans*	Hardjo	Hardjoprajitno	20	Reference	Aus& KIT
*L. interrogans*	Icterohaemorrhagiae	RGA	17	Reference	Aus& KIT
*L. interrogans*	Kenniwicki	LT1026	37	Reference	KIT
*L. interrogans*	Kuwait	136/2/2	26	Reference	MORU
*L. interrogans*	Lai	Lai	1	Reference	GenBank†
*L. interrogans*	Pomona	Pomona	37	Reference	Aus& KIT
*L. interrogans*	Portlandvere	MY1039	37	Reference	ND
*L. interrogans*	Schueffneri	Vleermuis90C	3	Reference	Aus
*L. interrogans*	Sumneri	Sumner	7	Reference	Aus& KIT
*L. interrogans*	Valbuzzi	Valbuzzi	61	Reference	Aus& KIT
*L. interrogans*	Autumanlis	3	34	Thailand	MORU
*L. interrogans*	Autumnalis	86	34	Thailand	MORU
*L. interrogans*	Autumnalis	L0020	34	Thailand	MORU
*L. interrogans*	Autumnalis	L0661	34	Thailand	MORU
*L. interrogans*	Autumnalis	L1151	34	Thailand	MORU
*L. interrogans*	Autumnalis	UT227	34	Thailand	MORU
*L. interrogans*	Autumnalis	548	34	Thailand	MORU
*L. interrogans*	Autumnalis	729	34	Thailand	MORU
*L. interrogans*	Autumnalis	LP101	22	Thailand	MORU
*L. interrogans*	Bataviae	L1111	42	Thailand	MORU
*L. interrogans*	Bataviae	UT229	46	Thailand	MORU
*L. interrogans*	Bataviae	UT234	46	Thailand	MORU
*L. interrogans*	Medanensis	L0448	46	Thailand	MORU
*L. interrogans*	Medanensis	L0887	46	Thailand	MORU
*L. interrogans*	Medanensis	L0941	46	Thailand	MORU
*L. interrogans*	Pomona	UT364	38	Thailand	MORU
*L. interrogans*	Pyrogenes	UD009	37	Thailand	MORU
*L. interrogans*	Pyrogenes	L0443	49	Thailand	MORU
*L. interrogans*	Pyrogenes	L0374	49	Thailand	MORU
*L. interrogans*	Unknown	654	33	Thailand	MORU
*L. interrogans*	Unknown	M04	34	Thailand	MORU
*L. interrogans*	Unknown	M08	34	Thailand	MORU
*L. interrogans*	Unknown	UT126	40	Thailand	MORU
*L. interrogans*	Unknown	L1085	42	Thailand	MORU
*L. interrogans*	Unknown	L0996	46	Thailand	MORU
*L. interrogans*	Unknown	UT053	46	Thailand	MORU
*L. interrogans*	Unknown	M10	49	Thailand	MORU
*L. interrogans*	Unknown	L1207	26	Thailand	MORU
*L. kirschneri*	Grippotyphosa	Moskva V	110	Reference	KIT
*L. kirschneri*	Mozdok	5621	117	Reference	KIT
*L. kirschneri*	Ratnapura	Wumalasena	116	Reference	KIT
*L. kirschneri*	Tsaratsovo	B 81/7	115	Reference	KIT
*L. kirschneri*	Vanderhoedeni	Kipod 179	110	Reference	KIT
*L. kirschneri*	Grippotyphosa	UT130	68	Thailand	MORU
*L. kirschneri*	Unknown	M06	68	Thailand	MORU
*L. kirschneri*	Unknown	M07	71	Thailand	MORU

# STs are not shown for the 6 loci scheme because this is not supported by a MLST website, and allelic numbers, profiles and STs have not been assigned to the sequence data.

*MORU, Mahidol-Oxford Tropical Medicine Research Unit, Bangkok, Thailand (MORU); KIT, KIT Biomedical Research, WHO/FAO/OIE Collaborating Center for Reference & Research on Leptospirosis, Amsterdam, Netherlands; Aus, WHO/FAO/OIE Collaborating Center for Reference & Research on Leptospirosis, Brisbane, Australia. Isolates from two different sources were identified using one of two MLST schemes only.

**†:** in silico analysis was performed on this isolate.

### Genotyping

All isolates were evaluated using both genotyping schemes [39],[40]. The MLST scheme described by Thaipadungpanit et al. (2007), is based on *pntA, sucA, pfkB, tpiA, mreA, glmU and fadD* [39], and the scheme described by Ahmed et al. (2006) is based on *adk, icdA, secY, rrs2, lipL41, and lipL32* [40]. The terms 7L and 6L have been adopted throughout to refer to the 7 and 6 gene schemes, respectively. No modifications were made to the published primers or cycling conditions of 7L. Table 2 lists the primer pairs used for 6L. Four of the 12 primers (*adk-*F, *adk-*R, s*ecY-*R and *icdA-*R) were modified compared with the published 6L scheme, and used in a repeat PCR reaction in the event that the original primers failed to generate an amplicon. Cycling conditions were as described previously for 6L, with the exception that reactions using the four new 6L primers had a reduced annealing temperature of 54°C. Sequence data were edited using SeqMan software contained within the DNASTAR package (DNASTAR Inc., Wisconsin, USA). The region of sequence used to define each locus of 7L was as described previously [39], but the region used to define each locus of 6L was altered as follows. Three loci (*secY*, *lipL32* and *lipL41*) were changed because the published PCR product and the region of sequence used to define the locus were either identical (*secY* and *lipL32*) or different by just two bp [40]. This meant that we were unable to obtain high quality sequence traces for the first 10–20 bases of the amplicon, and so trimmed the sequence in frame by approximately 20 bp at either end for all three genes. The other 3 published loci of 6L (*adk*, *icdA* and *rrs2*), were trimmed by one or two bases to put them in frame, which simplifies the analysis of synonymous and non-synonymous substitutions. The sequence start and end points for the 6 loci of 6L are shown in Table 2.

**Table 2:** Primers for 6 locus genotyping scheme used during this study [39].

Gene	Published primers (5′- 3′)	New primers (5′- 3′)	Location of sequence used to define MLST locus#	Size of MLST locus (bp)
*adk*	F-gggctggaaaaggtacacaa	F-acattatcttcatgggacctcc	3458789–3458361	429
	R-acgcaagctccttttgaatc	R-ttacacaagctccctttgaat		
*icdA*	F-gggacgagatgaccaggat		3980926–3980372	555
	R-ttttttgagatccgcagcttt	R-cttttttgagatctccggcttt		
*lipL32*	F-atctccgttgcactctttgc		1667072–1666641	432
	R-accatcatcatcatcgtcca			
*lipL41*	F-taggaaattgcgcagctaca		3603644–3604120	477
	R-gcatcgagaggaattaacatca			
*rrs2*	F-catgcaagtcaagcggagta		1862535–1862984	450
	R-agttgagcccgcagttttc			
*secY*	F-atgccgatcatttttgcttc		3459402–3458902	501
	R-ccgtcccttaattttagacttcttc	R-ccttcctttaattttagactttttc		

The alleles at each of the 7L loci were assigned and the sequence type (ST) defined using the publically accessible *Leptospira* MLST website (http://leptospira.mlst.net/). Allelic numbers, profiles and STs were not generated for the 6L data.

### Sequence analysis

Sequence alignment, nucleotide diversity and reconstruction of phylogenetic trees were performed using Molecular Evolutionary Genetics Analysis (MEGA) software version 4.0 [41]. Mean pairwise distances (p distance) were calculated using the Kimura Two Parameter nucleotide substitution model. Synonymous (dS) and non-synonymous (dN) nucleotide substitutions were calculated based on the Modified Nei-Gojobori method with Jukes Cantor correction using MEGA 4. Neighbor joining trees were reconstructed based on concatenated sequences of each scheme using the Kimura Two-Parameter substitution model. Gene order of the concatenated sequences were *glmU*, *pntA*, *sucA*, *fadD*, *tpiA*, *pfkB*, and *mreA* for 7L, and *adk, icdA, lipL32, lipL41, rrs2, and secY* for 6L. Discriminatory ability (D value) and 95% confidence intervals (CI) were estimated as described previously [42], [43]. These values were verified using the LIAN web tool housed on pubmlst.org [44]. A sliding window analysis of within- and between-species variation was carried out using DNAsp v. 5.0 [45]. An initial “window” of 400-bp was selected, as this is roughly equivalent to a single allele. The first window was thus from base 1 to base 400 of the concatenated sequence. From this we took each species in turn and calculated the average number of nucleotide differences per site over all pairwise comparisons (π), to give the within species polymorphism. Similarly, we calculated the number of fixed differences between species (substitutions) per site to gauge the divergence between *L. interrogans* and *L. kirschneri*. The window region was then moved along 50-bp and these parameters recalculated. GenBank accession numbers of 6L generated sequences are JF509178–JF509357.

## Results

### Discriminatory power of the two genotyping schemes

A total of 48 strains and isolates belonging to *L. interrogans* (n = 40) and *L. kirschneri* (n = 8) were included in this study, of which 17 were reference strains and 31 were clinical isolates – further referred to as strains (Table 1). Nine strains had been evaluated previously by both schemes [39], [40], and 39 strains typed previously by only one of the two schemes were typed by the other scheme during this study. Two strains (a Thai clinical isolate strain L1207 of unknown serovar and a reference strain of serovar Kuwait strain 136/2/2) could not be typed using 6L as both had a deletion of three nucleotides in the *lipL32* sequence. These two strains were excluded from further analysis.

7L resolved the 46 strains into 21 STs, shown in Table 1. 6L data were analysed off line, and the alleles at the six loci given arbitrary allelic numbers to construct an allelic profile and determine the number of genotypes. This demonstrated a total of 30 genotypes (data not shown). Overall levels of diversity (D) were comparable for the 7L and 6L schemes (92.0 (95% CI 87.5–96.5) and 93.5 (95% CI 88.6–98.4), respectively). The discriminatory ability per locus ranged from 59% (*sucA*) to 87% (*glmU* and *mreA*) for 7L and 66% (*rrs2*) to 92% (*secY*) for 6L (Table 3). All D values were verified using the LIAN web tool housed at pubmlst.org and found to be identical to the values shown. The majority of alleles of both schemes were species specific (that is, found in either *L. interrogans* or *L. kirscheri* but not both). There were three exceptions where alleles were found in both species, as follows: 7L, allele 1 of *sucA*; 6L, one allele of *lipL32* and one allele of *rrs2*.

**Table 3:** Discriminatory ability of two genotyping schemes and their respective loci.

	Number of alleles	p-distance#	dN/dS*	Discriminatory ability (%) (95% confidence intervals)
**7 loci scheme (21 STs)**				92.0 (87.5–96.5)
*glmU*	11	2.30%	0.073	86.9 (82.9–90.8)
*pntA*	11	3.60%	0.012	64.3 (49.0–79.5)
*sucA*	7	4.70%	0.007	59.3 (45.2–73.5)
*fadD*	7	4.00%	0.066	76.3 (69.1–83.5)
*tpiA*	10	6.10%	0.093	84.7 (79.1–90.4)
*pfkB*	14	4.70%	0.048	83.4 (76.0–90.7)
*mreA*	12	4.20%	0.007	86.9 (82.1–91.6)
Concatenated sequence of 6 loci (2,844 nt)		3.60%		
**6 loci scheme (30 genotypes)**				93.5 (88.6–98.4)
*adk*	10	6.70%	0.057	70.2 (57.2–83.2)
*icdA*	12	2.50%	0.022	74.8 (62.7–86.8)
*lipL32*	7	0.50%	0.154	71.9 (62.3–81.5)
*lipL41*	7	2.70%	0.01	81.9 (77.4–86.5)
*rrs2*	6	0.40%	ND	66.3 (58.1–74.4)
*secY*	20	5.50%	0.019	91.8 (87.4–96.2)
Concatenated sequence of 7 loci (3,165 nt)		2.30%		

# p distances were estimated based on the Kimura Two Parameter nucleotide substitution model.

*dN/dS were estimated based on the Modified Nei-Gojobori Method with Jukes Cantor correction using MEGA 4. The values shown represent a combined value for *L. interrogans* and *L. kirschneri*. dN/dS was not estimated for *rrs2* as this does not encode a protein.

### Nucleotide diversity of genetic loci

Overall nucleotide diversity (based on concatenated sequences) for the 46 isolates was 3.6% and 2.3% for 7L and 6L, respectively (Table 3). The diversity within *L. interrogans* was lower than that within *L. kirschneri* (0.5% and 1.1% for 7L, and 0.4% and 0.8% for 6L, respectively). Table 3 also details the nucleotide diversity by locus. This ranged from 3.6% to 6.1% for 7L, and 0.5% to 6.7% for 6L. The lowest diversity was observed for *lipL32* and *rrs2* of 6L. The dN/dS ratios calculated for each locus indicated that none were under positive selection (that is, all values were lower than 1) (Table 3).

**Figure 1:**
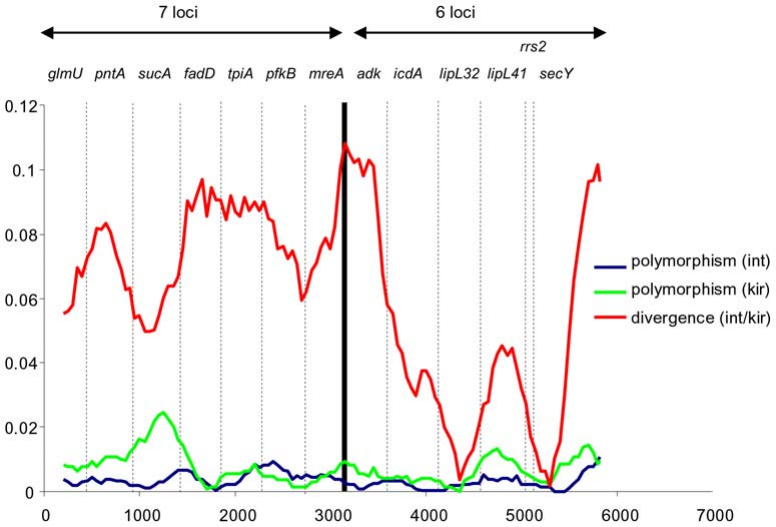
Sliding window analysis of concatenated sequence of all 13 loci. Sliding window analysis of concatenated sequence of all 13 loci, carried out using DNAsp v 5 using a window size of 400-bp, a step size of 50-bp, and points based on the mid-point of each window (i.e. the first point is at position 200). The names of the individual loci are shown. Three plots are given to represent the level of polymorphism within each of the two species, and the level of diversity between them. In terms of the within species variation, there is little difference between the two schemes and both point to generally higher levels of variation within *L. kirschneri* than *L. interrogans*. However, there are two loci used in the 6L scheme that are highly conserved between species (*lipL32* and *rrs2*), which means that in general the 7L scheme provides better between-species resolution.

A sliding window analysis of the concatenated sequences was performed to provide a visual comparison of the degree of polymorphism within both species, and the level of divergence between them. This revealed a generally higher level of variation within *L. kirschneri* compared to *L. interrogans*, particularly at *sucA* (7L) and to a lesser extent *lipL41* (6L), although the sample size for the former species was very small (n = 8) (Figure 1). This analysis confirmed that the degree of within species polymorphism showed very little difference between the 7L and 6L scheme. However, 7L tended to provide better resolution between species, which was largely accounted for by the low level of divergence for *lipL32* and *rrs2* of 6L.

### Relatedness of *Leptospira* spp. inferred from the two genotyping schemes

**Figure 2:**
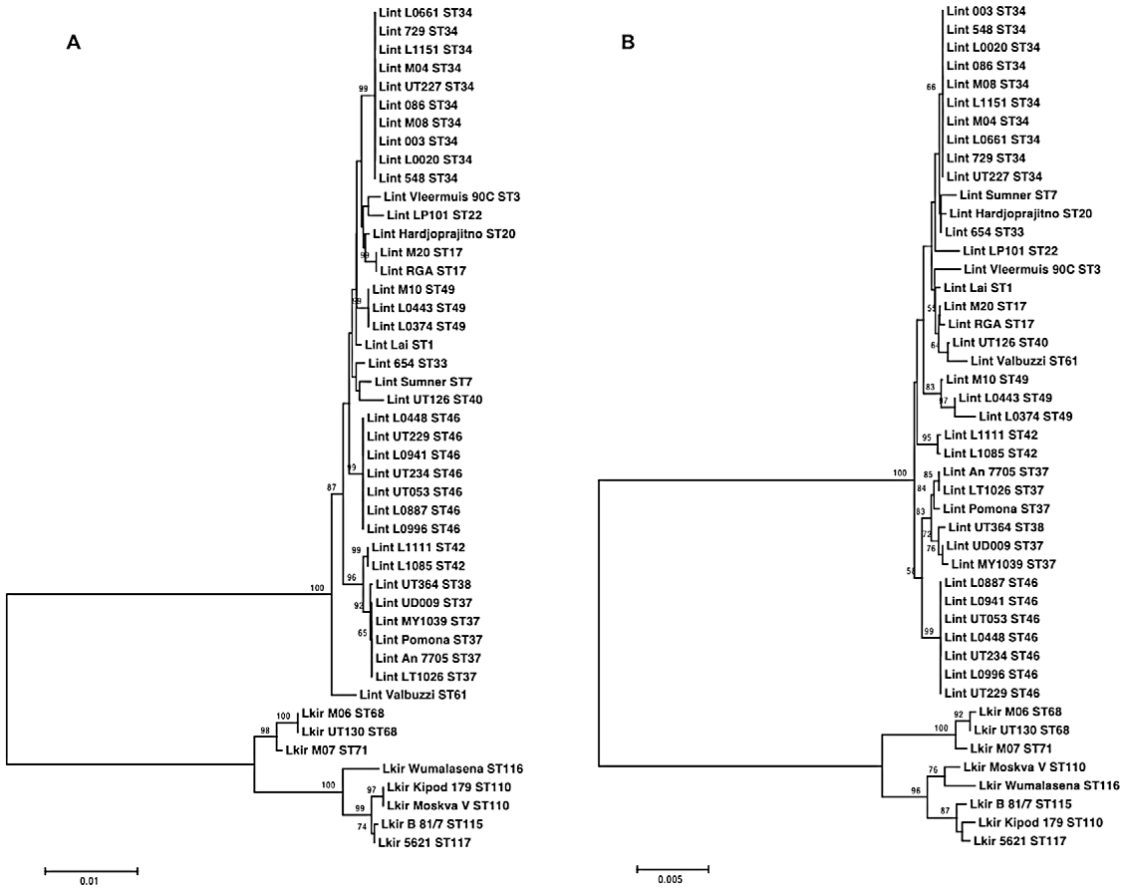
Neighbor joining trees of the 7L scheme and the 6 loci scheme. Neighbor joining trees reconstructed based on concatenated sequences of the 7L scheme (3,165 bp) (A), and the 6 loci scheme (2,844 bp) (B). Each bacterial strain is labeled by the following string: abbreviation of species name (Lint- *L. interrogans*, Lkir- *L. kirschneri*), strain name, and (for the 7L scheme only) sequence type (ST).

Neighbor joining trees were reconstructed for 7L and 6L based on the concatenated sequences of their respective loci (Figure 2). Both trees showed two distinct groups corresponding to *L. interrogans* and *L. kirschneri*. There were also several obvious similarities within *L. interrogans* between the two trees. For example, the clonal structure of ST34 and ST46 as defined by 7L was maintained by 6L. A common finding, however, was that 6L had a tendency to split single STs as defined by 7L into closely related clusters. For example, the three isolates designed as ST49 by 7L were split into three different genotypes by 6L. Further examples of splitting of a clone by the 6L scheme were 7L ST42, ST37, ST68 and ST17. A number of discrepancies were noted between the two trees. Two strains of *L. kirschneri* (strains Moskva V and Kipod 179) were designated by 7L as ST110, but these were resolved into different genotypes by 6L. These two strains differed by 9 nucleotides over 3 loci, with *secY* accounting for 7 of these. A difference was also noted for *L. interrogans* strain 654 (a Thai clinical isolate), which was closely related to *L. interrogans* strain Hardjoprajitno by 6L (differing by only 1 nucleotide), but was more distantly related by 7L (differing by 11 nucleotides over 6 loci).

## Discussion

The authors of this paper include representatives of the scientific groups that reported two independent genotyping schemes for *Leptospira* spp. Here, we provide the scientific community with the findings of a study that compared and contrasted the two schemes, together with a discussion of the practical aspects related to undertaking each.

The two schemes are unrelated and different by design. 7L was founded on a conventional strategy for MLST of selecting 7 housekeeping genes that were distributed around the genome and were not under positive selection. The design of 6L varied from this in that 6 loci were selected from different functional categories. For example, *lipL41* and *lipL32* encode surface expressed proteins that would be expected to be under positive selection as a result of being immunogenic and a target for the host response. At the other end of the spectrum, *rrs2* is one of two 16S rRNA genes that would be predicted to be highly conserved.

Contrary to our expectations, we did not find that any of the 6L genes were under positive selection. More genotypes were resolved by 6L than by 7L, in part a function of the high number of alleles for *secY*. Analysis of genetic diversity indicated that there was little difference in within-species variation difference between the two schemes, both pointing to generally higher levels of variation within *L. kirschneri* than *L. interrogans*. The conserved nature of two loci used in 6L (*lipL32* and *rrs2*), resulted in the finding on sliding window analysis that 7L provided better between-species resolution. Interestingly we noted that *rrs2* of 6L showed a higher D value than the housekeeping gene *sucA* of 7L. Although this is an exception to the general rule that housekeeping metabolic genes provide more discrimination than conserved genes such as those encoding ribosomal RNA, such an observation is not unprecedented [46].

6L has been applied to six pathogenic *Leptospira* spp. [40], which compares favorably with 7L which was designed for the two closely related species *L. interrogans and L. kirschneri*. However, this disadvantage of 7L will be resolved within the next 12 months; the scheme has already been extended to *L. borpetersenii* (manuscript in preparation), and the laboratory work to extend this to all pathogenic species is now completed. These improvements will be made publicly available by the end of 2011.

Conversely, the 6L scheme does not conform to the original concept of MLST as it includes a non-housekeeping gene (*rrs2*), and genes that encode cell surface proteins. Furthermore, the sequence start and stop sites used to define the allele for each locus were not provided in the original description of 6L scheme and so could not be performed based on the published methodology alone, although these have been detailed in this study. Minor changes were necessary to the start and stop sites, but we think it unlikely that this led to a change in the performance of the scheme.

The 6L scheme is not associated with a publically accessible website that allows an investigator to compare new data with existing sequence data. 6L has recently been applied to an extended set of strains and isolates (n = 271) encompassing a wide diversity of hosts and geographic regions [47], providing a rich source of sequence data that has been released into the public domain (GenBank). Comparative phylogenetic analysis by individual investigators will require downloading and storage of these data. In contrast, a website for 7L was launched at the time of publication and is regularly maintained and curated. At least one representative of each ST is recorded in a downloadable spreadsheet, providing a mechanism by which a picture of global bacterial diversity can be developed over time. This is easy to use, provides tools for comparison of a given strain with all of the other strains in the database, is more suited to investigators with limited phylogenetic training and experience, and so has the power to reach a wider audience.

In conclusion, we have provided detailed comparisons of two major genotyping schemes for *Leptospira* spp., and have described their advantages and disadvantages. 7L complies with the philosophy of MLST (housekeeping genes only supported by website), but will not be ready for use for the study of all pathogenic *Leptospira* spp. until the end of 2011. In the meantime, a bioinformatics analysis of the discriminatory power of 4 genes (three of which are not present in either scheme) as well as a new scheme with 7 loci both limited to *L. interrogans* and *L. kirschneri* have been reported [48], [49], adding further diversity to the tools available for the phylogenetic study of *Leptospira* spp. There is a pressing need for consensus within the leptospirosis community as to the preferred genotyping scheme, an essential step if the wealth of knowledge gathered for other bacterial species based on detailed analysis within a single scheme is to be replicated for *Leptospira* spp. Both schemes contain highly discriminative and less discriminative loci. While it is feasible to formulate a consensus MLST combining the most discriminative housekeeping genes from both schemes, we have resisted the temptation of presenting an interim scheme that has not been extensively validated. Instead, we aim to expedite the release of the 7L MLST scheme for all the major pathogenic species, and recommend its use for the study of the global epidemiology of pathogenic *Leptospira* spp.
